# Effects of Virtual Reality-Based Rehabilitation on Upper Extremity Function among Children with Cerebral Palsy

**DOI:** 10.3390/healthcare8040391

**Published:** 2020-10-10

**Authors:** Hyun Jung Chang, Kyo Hun Ku, Young Sook Park, Jin Gee Park, Eun Sol Cho, Jae Sam Seo, Chang Woo Kim, Se Hwi O

**Affiliations:** Department of Physical Medicine and Rehabilitation, Samsung Changwon Hospital, Sungkyunkwan University School of Medicine, Changwon 51353, Korea; reh.chj@gmail.com (H.J.C.); jijibaeheiwon@daum.net (Y.S.P.); jingee00@naver.com (J.G.P.); orange200000@naver.com (E.S.C.); j3likeu@naver.com (J.S.S.); bejimil007@naver.com (C.W.K.); oskdoh@naver.com (S.H.O.)

**Keywords:** virtual reality, cerebral palsy, upper extremity, occupational therapy, activities of daily living

## Abstract

Background: Deterioration in upper extremity function has been a common problem among children with cerebral palsy (CP). The present study evaluated the effects of virtual reality (VR)-based rehabilitation combined with conventional occupational therapy (COT) on upper extremity function and caregiver assistance among children with CP. Methods: Medical records of 17 children with CP who regularly participated in a rehabilitation program at Samsung Changwon Hospital were retrospectively reviewed. Ten children received VR-based rehabilitation, which utilized RAPAEL Smart Kids and video games combined with COT. Seven children received COT alone, which was provided by a trained occupational therapist and focused on their upper extremities. Clinical outcomes were determined using the Quality of Upper Extremity Skills Test (QUEST) and Pediatric Evaluation of Disability Inventory (PEDI), which were administered before and 8 weeks after the first intervention session. Results: The smart glove (SG) group showed significant improvements in all QUEST domains and five PEDI domains (*p* < 0.05), whereas the COT group showed a significant change only in total QUEST scores. A comparison between both groups revealed that the SG group had significantly greater improvements in five QUEST domains and two PEDI domains (*p* < 0.05). Conclusions: Our results suggest that VR-based rehabilitation combined with COT may improve the upper extremity functions and decrease caregiver burden among children with CP.

## 1. Introduction

Cerebral palsy (CP) is a well-recognized neurodevelopmental condition that begins during early childhood and persists throughout the individual’s lifespan [1]. Motor disorders associated with CP are often accompanied by disturbances in sensation, perception, cognition, communication, and secondary musculoskeletal problems [2]. Moreover, several children with CP experience tightness or weakness in their arms and hand muscles, which can lead to structural changes resulting in long-term difficulty in performing day-to-day tasks, such as dressing, feeding, and playing [3].

There has been growing interest in developing interventions for children with CP based upon assistive technology. One such area of interest is the use of virtual reality (VR) in developing functional independence and rehabilitation of motor skills [4,5,6,7]. VR can replace evaluation or training in situations where real-world application is difficult. Rehabilitation therapy is repetitive by nature, and repetition tends to reduce a patient’s motivation. VR-based rehabilitation has many benefits over conventional occupational therapy (COT), such as interactivity and motivation [8]. By providing visual and auditory feedbacks, such as displaying gratifying messages in real-time (“great”, “very good”, etc.), children become motivated to exercise. Moreover, VR allows the trainee to simulate dangerous situations [9]. For instance, training for activities of daily living (ADLs), such as cutting vegetables with a knife when cooking, may be difficult for children from a safety standpoint. Instead, VR can be a safe and effective alternative for such a dangerous environment.

VR seems to be effective in elevating self-efficacy, volition, playfulness, and motor functioning of children with CP [10]. However, most studies on VR-based rehabilitation for the upper extremities were reported, and there was no study on treatments limited to forearm and wrist movements [11,12]. In addition, it has not been sufficiently evaluated whether VR-based rehabilitation affects the caregiver burden in children with CP.

The current study aimed to evaluate the effects of VR-based rehabilitation combined with COT on upper extremity function and caregiver assistance among children with CP compared to COT alone.

## 2. Materials and Methods

**Figure 1:**
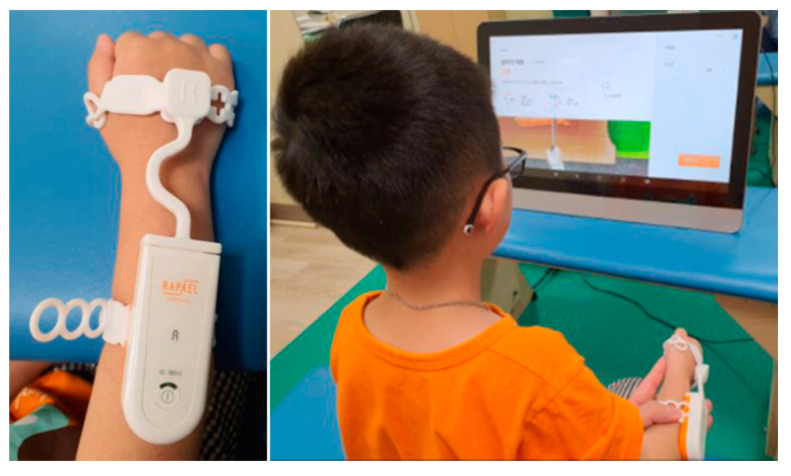
RAPAEL Smart Kids (Neofect Rehabilitation Solutions, Seongnam, South Korea).

This study retrospectively reviewed the medical records of 17 children with CP who participating in (Smart glove (SG) group) or waiting (COT group) for participation in the VR-based rehabilitation program at Samsung Changwon Hospital. The 17 patients who were selected received two treatments per week at our rehabilitation center. SG group received VR-based rehabilitation for 20 min combined with COT for 10 min per treatment session, whereas the COT group received only COT for 30 min per treatment session. VR-based rehabilitation combined with COT includes a range of motion (ROM), stretching exercise and RAPAEL Smart Kids (Neofect Rehabilitation Solutions, Seongnam, South Korea) therapy (Figure 1). COT included ROM exercise, stretching, and training for ADLs. Both groups performed a total of 16 treatments twice a week for eight weeks.

RAPAEL Smart Kids is a sensor-based rehabilitation training tool for children with neurological disorders. This wearable SG can sense wrist movement, which includes forearm supination/pronation, wrist flexion/extension, and wrist radial/ulnar deviation. Moreover, RAPAEL Smart Kids can be accompanied by 35 video games (e.g., fishing, sorting, driving, cooking, cutting, cleaning, painting, playing instruments, puzzle matching, etc.) designed to induce the targeted movement of the paretic wrist and forearm. Each game is categorized by cognition, active ROM, coordination, and timing, depending on the area of treatment. The game provided feedback in real-time and that induces high scores. Based on the judgment of the occupational therapist, the difficulty level and the treatment time of each game were adjusted to patients.

The use of patient data for research purposes was approved by the research ethics committee at the Samsung Changwon Hospital, Sungkyunkwan University School of Medicine, Korea (Approval No. SCMC 2020-08-012).

### 2.1. Outcome Measures

Clinical outcomes were measured using the Quality of Upper Extremity Skills Test (QUEST) and Pediatric Evaluation of Disability Inventory (PEDI). A professional occupational therapist administered the QUEST pre- and post-intervention. The patients’ parents completed the PEDI questionnaire pre- and post-intervention.

The QUEST was developed to overcome the limitations of currently available measures of hand function. This measure evaluates the quality of upper extremity function in four domains: dissociated movement, grasps, protective extension, and weight-bearing. Moreover, the QUEST designed children having neuromotor dysfunction with spasticity and had been validated for children aging 18 months to 8 years [13].

The PEDI [14] measures children’s functional abilities and performance in three domains (i.e., Self-care, Mobility, and Social function) on three dimensions (i.e., Functional skills, Caregiver assistance, and Modifications) and reflects aspects of the activity and participation dimension of the International Classification of Functioning, Disability, and Health for Children and Youth. Moreover, this measure has good evidence of reliability, validity, and responsiveness. The functional skills dimension consists of 197 items, each scored “unable” (0) or “capable” (1). The items are divided into three domains. The self-care domain (73 items) covers eating, grooming, dressing, and personal hygiene. The mobility domain (59 items) covers transfers, for example, in and out of bed, wheelchair and bathtub, indoor and outdoor locomotion, and stairs. The social function domain (65 items) covers communication, problem-solving, playing with peers, and safety. Each domain yields an aggregate score. The caregiver assistance and modifications dimensions consists of 20 items in the self-care (*n* = 8), mobility (*n* = 7), and social function (*n* = 5) domains. Each item of the caregiver assistance scale is rated from 5 (independent, no assistance is given or required) to 0 (total, child is completely dependent on assistance) [15]. This study used scores for the functional skills and caregiver assistance dimensions on a 0–100 scale, with higher score for the caregiver assistance dimension indicating lower level of support. The PEDI was translated into Korean before being administered to the parents.

### 2.2. Statistical Analysis

All statistical analyses were performed using SPSS 21.0 (SPSS Inc., Chicago, IL, USA). The Shapiro–Wilk test was used to describe the distribution of quantitative data. The Mann–Whitney test was used to compare baseline characteristics and initial functional states between both groups. Within-group changes without normal distribution were tested using the Wilcoxon signed-rank test and within-group changes with normal distribution were assessed using a paired *t*-test. Data were analyzed using analyses of covariance (ANCOVA), in which groups were compared according to post-intervention scores with pre-intervention scores as the covariate. A *p* value < 0.05 indicated statistical significance.

## 3. Results

No significant differences in average age, Gross Motor Function Classification System (GMFCS) level, Manual Ability Classification System (MACS) level, QUEST score, and PEDI score were observed between the two groups at baseline (Table 1).

The SG group showed significant improvement in dissociated movement, grasps, weight bearing, protective extension, and total QUEST scores after treatment (*p* = 0.027, 0.001, 0.012, 0.014, and 0.005, respectively). However, the COT group only showed a significant change in the total QUEST scores (*p* = 0.003; Table 2).

**Figure 2:**
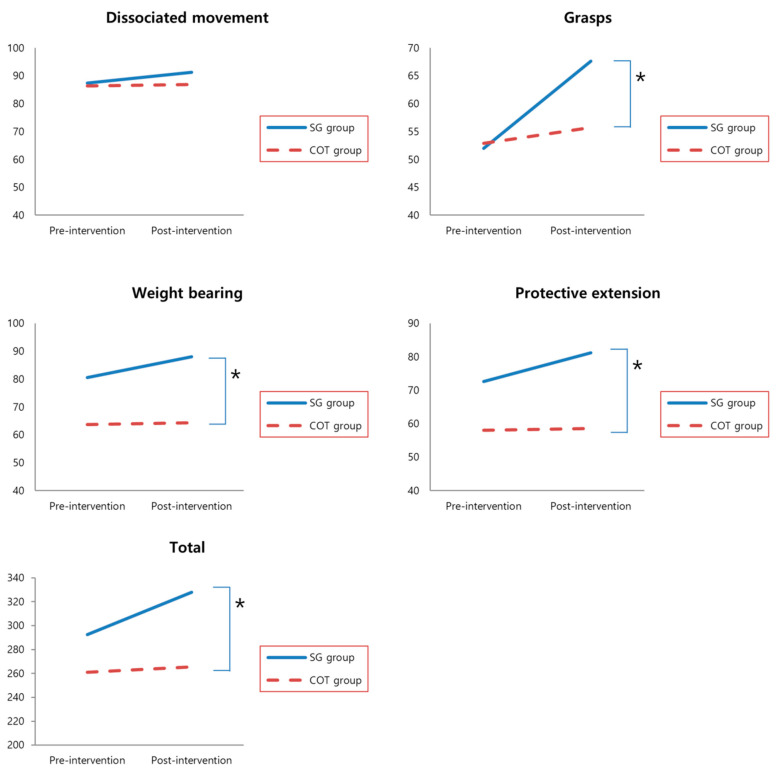
Mean of QUEST scores at pre- and post-intervention in the SG and COT groups. * Significant differences between groups (ANCOVA, *p* < 0.05). SG, Smart Glove. COT, conventional occupational therapy.

Pre- and post-intervention PEDI scores were obtained from the 17 children’s parents. Accordingly, the SG group demonstrated significantly improved scores in five PEDI domains (i.e., Self-care (*p* = 0.017), Transfer (*p* = 0.028), and Social function (*p* = 0.018) under the functional skills dimension and Self-care (*p* = 0.031) and Transfer (*p* = 0.022) under the caregiver assistance dimension) (Table 2). By contrast, no significant improvements in PEDI scores were found in the COT group. ANCOVA revealed significant differences between the groups in grasps (F = 9.75, *p* = 0.07), weight bearing (F = 10.04, *p* = 0.07), protective extension (F = 11.60, *p* = 0.004), total QUEST scores (F = 17.36, *p* = 0.001), and PEDI Transfer (F = 5.46, *p* = 0.035) (Figure 2) under caregiver assistance dimension. Other PEDI domains has pre-intervention scores * group effects.

## 4. Discussion

The current study aimed to evaluate the effects of VR-based rehabilitation combined with COT on upper extremity function and caregiver assistance among children with CP. Our results found that VR-based rehabilitation showed beneficial effects by improving upper extremity skills and functional skills among children with CP. To our knowledge, there has previously been no research to verify the effectiveness of caregiver burden along with the improvement of upper limb function and ADLs in the treatment of distal upper extremity using VR. 

A systematic review by Lucas et al. showed that 12-session, task-oriented interventions are best for improving children’s motor performance. Moreover, another study demonstrated that traditional therapeutic approaches applied to children were very beneficial for motor function [16]. It was appropriated that 8 weeks post-intervention assessment in our study.

As the period of rehabilitation increases, it can be boring to children because of the simple repetitive nature of rehabilitation. Motivation is considerably important in the rehabilitation process of CP given the prolonged duration needed to reach desired functional level [17]. VR technology allows therapists to motivate children during the rehabilitation process through the pleasure of gaming [18]. To increase functional independence in daily activities, VR provides opportunities for repetitive exercises and positive feedback of motor performance [19].

In addition to the movement of wrist and forearm, which were the main treatment areas in this study, we also observed improvements in the movement control of shoulder and hand. The SG group showed improvement in all domain and total score of QUEST, while the COT group showed improvement only in total score. This is perhaps due to the incorporation of accurate repetitive movements that require coordination and visual tracking in VR games. For correct movement, the participants were also required to control the isolated movement of the wrist and forearm and the control of other joints. These isolated movements can be difficult for children with CP because of their upper extremities that are using the synergic pattern of flexion-pronation. Chen et al. [20] suggested that repeated engagement in VR games can improve hand–eye coordination among children, which is reflected by the visual motor skill score. Visual feedback from VR games and practice of repeated movements through the therapist’s verbal commands allowed children to analyze and correct errors in their movements, which were reflected in their fine motor coordination.

Choi et al. [21] reported that commercial gaming-based VR movement therapy was as effective as COT for recovery of upper extremity motor function and ADL among patients suffering from stroke. However, the current study found that VR-based rehabilitation promoted better improvements in functional upper extremity activity and ADLs than COT. Several hypotheses may help explain such findings. First, study of stroke patient was done on patients in subacute stage. Therefore, the potential of functional recovery was higher than that of children with CP, which is a chronic stage. Second, the patient who is the subject of our study had been undergoing COT for a long time. Therefore, the motivation for COT could have reduced. VR-based rehabilitation could be a new challenge, which can lead to increased motivation. 

One of the strongest predictors for ADLs limitations and participation restrictions among children with CP is their limited ability to manipulate objects with their hands [22]. A systematic review [23] revealed that hand use development among children with bilateral CP was among the most challenging concerns during rehabilitation. To develop hand function, utilizing a single type of treatment alone may be insufficient, especially among those needing long-term follow-up. Accordingly, the results presented herein showed that COT alone had a positive effect on only total QUEST score. However, in the SG group, there was more impact in all QUEST domains, and significant differences between groups in grasps, weight-bearing, protective extension, and total QUEST scores. Although the applied games were mainly about movements of wrist and forearm, there was an improvement in overall upper extremity functions. The present study has several limitations worth noting. First, this was a non-blinded retrospective study. The therapist who treated evaluated QUEST. In addition, it is possible that the PEDI questionnaire was obtained by the caregiver of patients receiving a new type of VR-based rehabilitation and affected the results. Second, this study may have limited significance due to its small sample size. Furthermore, a longer period of follow-up assessments might be necessary after VR-based rehabilitation to determine whether improvements in the upper extremity functions and caregiver burden are temporary or sustained. A larger sample size and longer follow-up period could help elucidate the therapeutic effects of VR-based rehabilitation more definitively. Third, no significant differences in GMFCS and MACS level had been observed between groups. However, the number of patients with higher GMFCS and MACS levels in COT group. In this study, the level of treatment was adjusted by the therapist according to the function of each patient, possibly resulting in fewer functional improvements. In some QUEST and PEDI domains, pre-intervention scores were higher in the SG group than in the COT group. Although the SG group showed more significant impact in comparison to the within group, it could have been due to an interaction based on higher pre-intervention scores. Therefore, the interaction effect of pre-intervention score with group was analyzed. The interaction effect was observed in the PEDI domains, except for the transfer domain under the caregiver assistance dimension. Further blinded randomized controlled trials should be performed to compare participants according to the baseline severity of upper extremity impairments.

## 5. Conclusions

The present study showed that VR-based rehabilitation with COT among children with CP might have better positive effects on upper extremity function, performance, and caregiver assistance than COT alone. In addition, such improvements in upper extremity functions and repeated practice of ADLs have resulted in reducing the burden on caregivers. This demonstrates that VR-based rehabilitation can increase compliance with children with CP through the inducement of interest, thereby increasing the effectiveness of treatment. Therefore, this study is meaningful in the clinical application of VR-based rehabilitation programs in children with CP.

## Figures and Tables

**Table 1:** Baseline demographic characteristics of the participants.

Characteristic	SG Group (*n* = 10)	COT Group (*n* = 7)	*p*
Age (year)	6.08 ± 1.77	4.88 ± 1.15	0.169
Gender			
Male	7	5	
Female	3	2	
Affected hand			
Right hand	7	1	
Left hand	2	2	
Bilateral hand	1	4	
GMFCS level	1.10 ± 0.32	2.28 ± 1.38	0.088
I	9	3	
II	1	1	
III	0	1	
IV	0	2	
MACS level	1.60 ± 0.70	1.42 ± 0.78	0.601
I	5	5	
II	4	1	
III	1	3	
IV	0	0	
QUEST domains			
Dissociated Movement	87.40 ± 12.15	86.28 ± 6.58	0.230
Grasps	52.00 ± 20.07	52.85 ± 14.83	0.813
Weight Bearing	80.60 ± 15.32	63.71 ± 20.25	0.055
Protective Extension	72.60 ± 16.28	58.00 ± 9.10	0.07
Total	292.60 ± 56.97	260.86 ± 44.67	0.27
PEDI domains			
Functional Skills			
Self-care	57.60 ± 12.16	49.29 ± 8.10	0.07
Transfer	52.60 ± 3.98	40.14 ± 14.80	0.109
Social function	57.00 ± 5.94	49.43 ± 10.13	0.07
Caregiver Assistance			
Self-care	27.00 ± 11.96	17.14 ± 11.48	0.23
Transfer	29.60 ± 4.53	21.29 ± 13.09	0.23
Social function	22.30 ± 2.36	17.43 ± 9.22	0.364

Values are presented as mean ± standard deviation. GMFCS, Gross Motor Function Classification System. MACS, Manual Ability Classification System. QUEST, Quality of Upper Extremity Skills Test. PEDI, Pediatric Evaluation of Disability Inventory. SG, Smart Glove. COT, conventional occupational therapy.

**Table 2:** Descriptive statistics for the pre- and post-intervention QUEST and PEDI scores.

Variable	SG Group		*p*	COT Group		*p*
	Pre	Post		Pre	Post	
QUEST Domains						
Dissociated movement	87.40 ± 12.15	91.2 ± 9.90	0.027 ^a^	86.28 ± 6.58	86.86 ± 6.82	0.172 ^b^
Grasps *	52.00 ± 20.07	67.60 ± 20.08	0.001 ^b^	52.85 ± 14.83	55.71 ± 14.76	0.059 ^a^
Weight bearing *	80.60 ± 15.32	88.00 ± 13.30	0.012 ^a^	63.71 ± 20.25	64.28 ± 19.91	0.356 ^b^
Protective extension *	72.60 ± 16.28	81.20 ± 12.87	0.014 ^b^	58.00 ± 9.10	58.57 ± 10.18	0.356 ^b^
Total *	292.60 ± 56.97	328.00 ± 51.99	0.005 ^a^	260.86 ± 44.67	265.42 ± 45.46	0.003 ^b^
PEDI Domains, Functional Skills Dimension						
Self-care	57.60 ± 12.16	63.50 ± 7.32	0.017 ^a^	49.29 ± 8.10	49.43 ± 8.36	0.356 ^b^
Transfer	52.60 ± 3.98	55.40 ± 3.41	0.028 ^a^	40.14 ± 14.80	40.14 ± 14.80	–
Social function	57.00 ± 5.94	61.00 ± 2.00	0.018 ^a^	49.43 ± 10.13	49.71 ± 10.14	0.356 ^b^
PEDI Domains, Caregiver Assistance Dimension						
Self-care	27.00 ± 11.96	32.10 ± 5.09	0.031 ^a^	17.14 ± 11.48	17.42 ± 11.25	0.172 ^b^
Transfer *	29.60 ± 4.53	30.90 ± 4.01	0.022 ^b^	21.29 ± 13.09	21.29 ± 13.09	–
Social function	22.30 ± 2.36	23.30 ± 1.70	0.168 ^a^	17.43 ± 9.22	17.57 ± 9.29	0.356 ^a^

Values are presented as mean ± standard deviation. QUEST, Quality of Upper Extremity Skills Test. PEDI, Pediatric Evaluation of Disability Inventory. SG, Smart Glove. COT, conventional occupational therapy. ^a^ Wilcoxon signed ranks test to compare between values of pre- and post-intervention values. ^b^ Paired *t*-test to compare between values of pre- and post-intervention values. * Significant differences between groups (ANCOVA, *p* < 0.05).
